# Sentiment Analysis of Autologous Breast Reconstruction Using Natural Language Processing and Deep Learning

**DOI:** 10.1093/asjof/ojaf145

**Published:** 2025-12-24

**Authors:** Melanie J Wang, Arman J Fijany, Cole A Holan, Gowri Warikoo, Jorys Martinez-Jorge

## Abstract

**Background:**

Use of autologous flaps for postmastectomy reconstruction has increased. Public forums offer large-scale patient narratives that can be analyzed with natural language processing (NLP) models, such as RoBERTa.

**Objectives:**

This study aims to assess patient-reported sentiment and emotions toward autologous reconstruction with deep inferior epigastric perforator (DIEP), transverse rectus abdominis myocutameous (TRAM), and latissimus dorsi (LD) flaps.

**Methods:**

Public reviews from RealSelf (Seattle, WA) referencing DIEP, TRAM, or LD flaps were collected. Two NLP models were applied: (1) a binary sentiment classifier (positive/negative) and (2) an emotion model scoring fear, sadness, anger, disgust, neutral, surprise, and joy; each review was labeled by its highest-scoring emotion.

**Results:**

Two hundred and twelve posts were analyzed: 153 DIEP, 20 TRAM, and 39 LD. DIEP had the highest mean positive sentiment (0.627); TRAM had the lowest (0.517). In the emotion model, “joy” was most common for DIEP (68/153; mean 0.389) and TRAM (7/20; mean 0.299). LD posts were most often “neutral” (9/39; mean 0.267).

**Conclusions:**

NLP of public patient reviews suggests DIEP flaps are the most discussed and are associated with more positive sentiment than TRAM or LD reconstructions. Emotion profiles indicate generally joyful narratives for DIEP, neutral for LD, and comparatively less positive for TRAM. This approach complements traditional outcomes research by capturing real-world patient experience at scale.

**Level of Evidence: 3 (Therapeutic):**

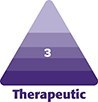

Breast cancer is a significant health challenge that affects ∼1 in 8 women during their lifetime.^1^ With advancements in screening, surgical techniques, systemic therapies, and radiation, the overall survival rates for breast cancer patients have improved markedly over recent decades.^2,3^ However, the experience of undergoing a mastectomy can have profound psychological and physical impacts, including body image concerns, emotional distress, and decreased quality of life.^4-6^ As a result, postmastectomy breast reconstruction has become a vital component of comprehensive cancer care, aimed at restoring physical appearance and supporting emotional well-being. Recent data indicate that between 40% and 60% of women who undergo mastectomy pursue some form of breast reconstruction, choosing from a variety of surgical options, including immediate, implant-based or autologous tissue procedures, as well as delayed procedures using tissue expanders.^7-9^ This growing trend underscores the importance of understanding patient preferences, satisfaction, and emotional responses related to different reconstructive techniques to optimize patient-centered care and shared decision making.

Despite the proliferation of various reconstruction techniques such as deep inferior epigastric perforator (DIEP), transverse rectus abdominis myocutaneous (TRAM), and latissimus dorsi (LD) flaps, the understanding of patient satisfaction and emotional experiences remains limited.^10^ Traditional methods of assessing patient outcomes rely on surveys and clinical interviews, which are often constrained by small sample sizes, response biases, and temporal limitations.^10,11^ These limitations hinder a comprehensive understanding of how patients perceive and emotionally relate to their reconstruction choices, leaving a gap in patient-centered insights that could inform clinical decision making.

The advent of online health communities and social media platforms offers a new avenue to explore patient perspectives at scale. In recent years, online healthcare forums and social media platforms have created new opportunities for large-scale analysis of patient-reported outcomes. One such platform, RealSelf (Seattle, WA), founded in 2006, is an online community that hosts patient ratings and reviews for cosmetic and reconstructive procedures. Patients can post reviews, updates, before-and-after photographs, and summaries of their overall satisfaction, often using a proprietary rating system of “worth it,” “not worth it,” or “not sure.” However, a majority of these reviews include a detailed block of text without selecting one of these formal ratings. This makes natural language processing (NLP), a less biased way to determine patient satisfaction. NLP, a subset of artificial intelligence, enables systematic extraction of insights from unstructured textual data, offering a novel approach to understanding patient satisfaction and experiences with various breast reconstruction modalities. The aim of the authors of this study is to leverage NLP and deep learning techniques to analyze patient sentiments expressed in publicly available reviews regarding DIEP, TRAM, and LD flaps, providing valuable insights to clinicians and patients alike.

## METHODS

### Data Collection

A cross-sectional study was conducted, utilizing publicly available data from the online healthcare forum RealSelf, which hosts user-generated content on cosmetic and reconstructive procedures. RealSelf hosts procedure-specific pages where patients describe their experiences and optionally assign a star rating. Patient reviews related to autologous breast reconstruction procedures were systematically collected in July 2024 for all reviews provided from January 2014 to July 2024. We focused on reviews that referenced autologous flap breast reconstruction, specifically DIEP, TRAM, and LD flaps. Because RealSelf does not collect structured preoperative patient-reported outcomes (eg, baseline BREAST-Q) or surgical indication (primary vs salvage), our outcomes reflect postoperative sentiment only. Reviews were selected based on keywords and tags associated with each procedure, and only reviews written in English with at least 20 words of procedure-related content were included to ensure sufficient detail for NLP analysis. Reviews consisting solely of brief comments (eg, “great surgeon” and “very happy”) without reference to the reconstruction experience were excluded. Two independent reviewers screened all reviews for inclusion, and disagreements were resolved by consensus to minimize bias. Reviews that included concurrent procedures (ie, liposuction) were excluded to reduce confounding. This approach allowed for a snapshot of patient perceptions and sentiments associated with each reconstruction type at a specific point in time.

We analyzed both the numeric star rating and the accompanying free-text narrative for each review. In addition to overall sentiment, we extracted prespecified content themes that commonly drive patient evaluations in breast reconstruction: aesthetic results (eg, symmetry, natural appearance, and size/volume), donor-site morbidity (abdominal bulge/weakness for DIEP/TRAM; shoulder function/animation deformity for LD), pain/recovery burden, scarring, complications/revisions (eg, infection, seroma, fat necrosis), surgeon communication/expectation management, care experience, and cost/insurance. Because this study used only publicly available, de-identified patient reviews, it did not require IRB approval.

### Data Processing

Textual data underwent standard NLP preprocessing, including tokenization, lowercasing, removal of stop words, and stemming. To ensure data quality, reviews with minimal content (<20 words) were excluded. Physician identifiers and portions of the review describing the provider's demeanor and character were excluded from the NLP analysis of the review. The dataset was then divided into training and validation sets, maintaining proportional representation of each procedure.

### Natural Language Processing Models and Analysis

Two advanced deep learning models were employed to analyze the textual data. The first was a sentiment analysis model based on a fine-tuned Bidirectional Encoder Representations from Transformers (BERT). This model scored each review on a scale from 0 to 1, with higher scores indicating more positive sentiment. The second was an emotion classification model utilizing a pretrained Robustly Optimized Bidirectional Encoder Representations from Transformers (RoBERTa) architecture. This multiclass classifier identified the predominant emotion expressed in each review, such as fear, sadness, anger, disgust, neutral, surprise, or joy, and assigned an emotion strength score from 0 to 1, reflecting the intensity of the detected emotion. The BERT-based sentiment model produced scores ranging from 0 to 1, where higher values reflect more positive sentiment. For classification, we set a 0.5 threshold: sentiment scores above 0.5 were labeled as positive, whereas scores 0.5 or below were classified as neutral or negative. This cutoff aligns with approaches in similar sentiment analysis studies, such as Lin et al, where 0.5 was used as the decision point between positive and negative sentiment.^11^ Together, these models provided a comprehensive understanding of both the general emotional tone and specific feelings conveyed in patient reviews.

### Statistical Analysis

Sentiment scores and emotion classifications were statistically analyzed to compare perceptions across the 3 reconstruction types. Descriptive statistics summarized the average sentiment and emotion scores.

## RESULTS

A total of 212 patient reviews were collected: DIEP flap reviews (*n* = 153, 72.2%), TRAM flap reviews (*n* = 20, 9.4%), and LD flap reviews (*n* = 39, 18.4%).

### Sentiment Analysis

The sentiment analysis, which studies the emotional tone behind each of the patient reviews, was first conducted on this data. Each review was scored on a scale of 0 to 1, with values closer to 1 correlating with an increased positive tone of the review. DIEP flap reviews received an average positive sentiment score of 0.627, compared with 0.517 for TRAM flaps and 0.267 for LD flaps (Table 1).

**Table 1.** Sentiment Scores by Reconstruction Type (DIEP, TRAM, and LD)

Reconstruction type	No. of reviews (%)	Positive sentiment score		Negative sentiment score	
		*n* (%)	Score	*n* (%)	Score
DIEP	153 (72.2)	122 (79.7)	0.627	31 (20.2)	0.126
TRAM	20 (9.4)	14 (70)	0.517	6 (30)	0.165
LD	39 (18.4)	32 (82.1)	0.267	7 (17.9)	0.137

Values Shown are Counts (*n*, %) and Mean Sentiment Scores (0-1). DIEP, deep inferior epigastric artery perforator; LD, latissimus dorsi; TRAM, transverse rectus abdominis myocutaneous.

### Emotion Classification

**Figure 1.**
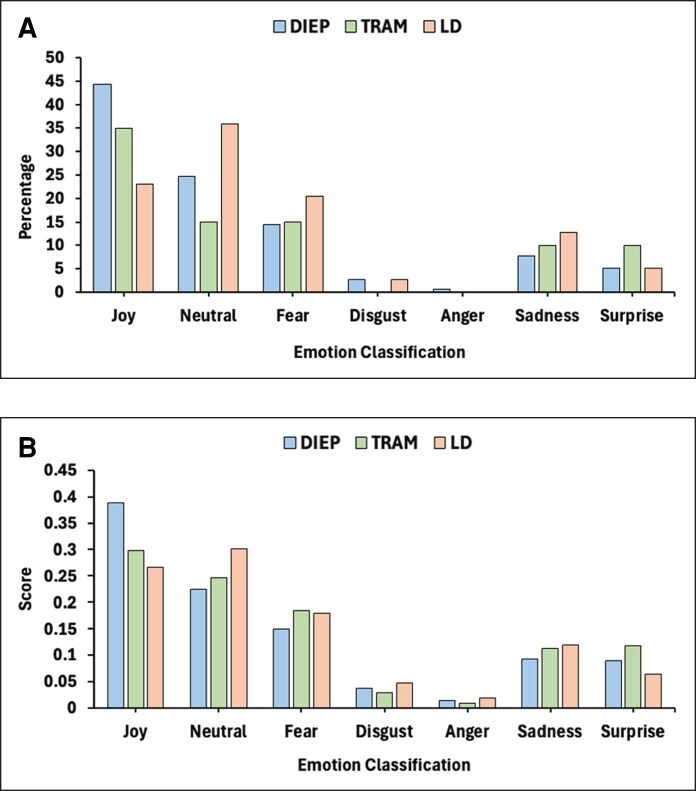
Emotion classification of patient reviews after autologous breast reconstruction. (A) The percentage of reviews for each emotion. (B) The mean intensity score (0-1). DIEP, deep inferior epigastric artery perforator; LD, latissimus dorsi; NLP, natural language processing; TRAM, transverse rectus abdominis myocutaneous.

The emotion-based model (Figure 1) then studied each review to identify the most correlated emotion with its respective breast flap. “Joy” was the most commonly displayed emotion in DIEP flaps with 68 of the 153 displaying such and an average score of 0.389 per review. “Joy” was also the primary emotion identified in TRAM flaps, but in this case, with only 7 of the 20 reviews corroborating this and a score of 0.299 per review. The primary emotion expressed in LD flaps was “Neutral” with 14 of the 39 expressing this and a score of 0.267 per review. DIEP flaps were the most positively perceived by patients. In the sentiment analysis, LD flaps were the least positively perceived; however, the emotion-based model shows that they were mainly associated with a neutral emotion, indicating indifference within patients receiving such procedures (Supplemental Table 1).

## DISCUSSION

DIEP flaps are associated with the most positive sentiment and strongest emotional approval among the 3 most used autologous breast reconstruction techniques, suggesting a potential preference among patients for procedures that balance aesthetic outcomes and muscle preservation. This finding reinforces the clinical observation that DIEP flaps are often regarded as the gold standard because of their favorable cosmetic results, minimal donor-site morbidity, and preservation of abdominal wall integrity.^12^ By analyzing patient-reported experiences from a public, anonymous platform using robust NLP tools, we offer novel, data-driven insights into the emotional and psychological dimensions of patient satisfaction beyond traditional clinical metrics.^13^ The use of RealSelf reviews as a data source reflects a growing trend in healthcare research to extract meaningful patient experience metrics from online platforms.^14^ Previous work has shown that although the relationship between online reviews and traditional patient satisfaction scores remains unclear, these reviews offer a rich repository of real-world patient sentiment and individualized feedback.^15,16^ Conventional tools, such as the BREAST-Q and BRECON-3, remain widely used for standardized outcomes assessment; however, they often lack the spontaneity, emotional granularity, and volume of online reviews.^17,18^ By leveraging NLP and deep learning, our analysis bridges this gap, capturing subtleties in emotional tone, including joy, neutrality, sarcasm, and dissatisfaction, at a scale not feasible with traditional surveys.

Patient satisfaction is inherently multifactorial, influenced by surgical outcomes, expectations, aesthetics, recovery experience, and provider interactions. This is particularly relevant in plastic surgery, where reconstructive and aesthetic goals often intersect. Previous studies have identified correlations between positive online reviews and variables such as surgeon demeanor, empathy, and aesthetic satisfaction.^19^ Our study builds on that literature by distinguishing between procedure-specific emotional profiles. Although DIEP flaps elicited the highest levels of joy and positivity, LD flaps demonstrated more neutral responses, suggesting neither high satisfaction nor strong discontent. In addition to higher average positive sentiment scores, DIEP flap reviews also demonstrated the poorest average negative sentiment scores when compared with TRAM and LD flaps. This distribution suggests that patient experience with DIEP flaps may be more polarized, with individuals perceiving outcomes as either highly favorable or less satisfactory, rather than moderate. This highlights the importance of setting appropriate expectations during preoperative counseling, as even procedures with high satisfaction rates may generate strongly divergent responses depending on individual patient experiences. Discussing these emotional nuances with patients can supplement provider preoperative counseling, thereby helping patients set realistic expectations, and aligning surgical choices with personal values.

In addition to the overall emotional profiles, our results demonstrate that fear appears elevated in patient reviews of LD flap reconstruction compared with DIEP and TRAM flaps. This is consistent with previous literature, which suggests that the functional consequences and unique risks of LD flap harvest (ie, impaired shoulder function, back discomfort, and donor-site morbidity) may contribute to increased anxiety regarding both immediate and long-term outcomes.^20^ Patients frequently report concerns related to mobility limitations, loss of upper body strength, and potential impairment in daily activities after LD flap procedures, which may manifest as heightened fear or apprehension in their narratives. Moreover, psychological distress may be exacerbated among patients who require LD flaps as salvage procedures or those with histories of axillary lymph node dissection and radiotherapy, groups shown to have distinct patterns of dissatisfaction and worry about postoperative function. These findings underscore the importance of comprehensive patient education and expectation management before surgery, as well as the potential value of targeted psychosocial support for those expressing elevated fear or anxiety. Addressing procedural risks, anticipated recovery limitations, and strategies for functional rehabilitation during preoperative counseling may mitigate fear and enhance patient satisfaction and adjustment after LD flap-based reconstruction. Thus, the observed trend of higher fear in LD flap reviews provides meaningful insight for clinicians aiming to optimize emotional outcomes and shared decision making in breast reconstruction.

Clinically, the authors of this paper emphasize the utility of integrating patient sentiment data into reconstructive decision making. Real-world patient narratives analyzed with NLP can serve as supplemental counseling tools, highlighting trends in satisfaction and anticipated emotional response. For example, patients considering TRAM flaps may benefit from understanding their slightly lower sentiment scores and emotional engagement compared with DIEP options. Meanwhile, those selecting LD flaps can be better prepared for more functionally driven outcomes with less emotionally charged responses. These insights not only empower patients to make more informed decisions but also enable providers to proactively address common emotional and physical concerns during the shared decision-making process. RealSelf narratives are complementary to validated patient reported outcome measures such as the BREAST-Q. Accordingly, our results are hypothesis generating and not prescriptive for flap selection.

Finally, the broader significance of this study lies in its demonstration of how artificial intelligence tools can augment traditional patient-reported outcome research.^21,22^ Unlike surveys, which can suffer from response bias and time limitations, platforms like RealSelf offer spontaneous, diverse, and emotionally rich data. Future research should build on these findings by incorporating multimodal data sources, validating sentiment trends across institutions, and integrating patient feedback loops into quality improvement frameworks. Because healthcare moves toward increasingly personalized and patient-centered models, combining surgical innovation with artificial intelligence will be essential in enhancing care quality and satisfaction outcomes.

This study is limited by its reliance on publicly available online reviews, which may not fully represent the broader patient population. The self-selected nature of online feedback introduces potential bias, because highly satisfied or dissatisfied patients are more likely to post reviews. The small sample sizes, particularly for TRAM and LD flaps, restrict the generalizability of findings. Because RealSelf does not capture structured preoperative patient reported outcomes or surgical indication, our analyses reflect postoperative sentiment only. Future studies should incorporate larger datasets and validate findings with prospective patient surveys or interviews, as well as incorporate multimodal data, such as images and video reviews, and expand to other social media platforms for a more comprehensive analysis. This study is also limited by its reliance on descriptive statistics without formal tests of statistical significance when comparing sentiment across reconstruction types. As a result, these findings should be interpreted with caution, and future research with inferential statistical analyses is warranted to more robustly validate these observed trends.

## CONCLUSIONS

The application of NLP and deep learning to patient-generated online reviews offers valuable insights into patient satisfaction and emotional responses to different autologous breast reconstruction techniques. DIEP flaps are generally perceived most positively, whereas LD flaps tend to evoke neutral sentiments. These findings can aid clinicians in personalized patient counseling and highlight the potential of artificial intelligence tools in understanding patient perspectives at scale. As reconstructive techniques evolve, integrating patient sentiment analysis into clinical decision making may enhance patient-centered care and improve overall satisfaction.

## Supplemental Material

This article contains supplemental material located online at https://doi.org/10.1093/asjof/ojaf145.

## Supplementary Material

ojaf145_Supplementary_Data
