# Supplementary material for: Sentiment Analysis of Autologous Breast Reconstruction Using Natural Language Processing and Deep Learning
Source: Aesthet Surg J Open Forum. 2025 Dec 24;7:ojaf145. doi: 10.1093/asjof/ojaf145 (PMC12728504; doi:10.1093/asjof/ojaf145)
Supplement: ojaf145_Supplementary_Data [file ojaf145_supplementary_data.docx]

**Supplementary Table 1.** Primary emotion identified on natural language processing assessment of patient reviews following DIEP, TRAM, and LD breast reconstruction.

| **Emotion** | | **DIEP 153** | **TRAM 20** | **LD 39** |
| --- | --- | --- | --- | --- |
| **Joy** | N (%) | 68 (44.4) | 7 (35.0) | 9 (23.1) |
|  | Score | 0.389 | 0.299 | 0.267 |
| **Neutral** | N (%) | 38 (24.8) | 6 (15.0) | 14 (35.9) |
|  | Score | 0.225 | 0.247 | 0.301 |
| **Fear** | N (%) | 22 (14.4) | 3 (15.0) | 8 (20.5) |
|  | Score | 0.150 | 0.185 | 0.180 |
| **Disgust** | N (%) | 4 (2.6) | 0 | 1 (2.6) |
|  | Score | 0.038 | 0.029 | 0.048 |
| **Anger** | N (%) | 1 (0.7) | 0 | 0 |
|  | Score | 0.015 | 0.009 | 0.020 |
| **Sadness** | N (%) | 12 (7.8) | 2 (10.0) | 5 (12.8) |
|  | Score | 0.093 | 0.113 | 0.119 |
| **Surprise** | N (%) | 8 (5.2) | 2 (10.0) | 2 (5.1) |
|  | Score | 0.090 | 0.118 | 0.065 |

DIEP: Deep Inferior Epigastric Artery Perforator; TRAM: Transverse Rectus Abdominis Myocutaneous; LD: Latissimus Dorsi.
